# Effect of Vitamin D3 on Mitochondrial Biogenesis in Granulosa Cells Derived from Polycystic Ovary Syndrome

**DOI:** 10.22074/ijfs.2020.6077

**Published:** 2020-07-15

**Authors:** Zahra Safaei, Shabnam Bakhshalizadeh, Mohammad Hossein Nasr Esfahani, Azadeh Akbari Sene, Vahid Najafzadeh, Mansoureh Soleimani, Reza Shirazi

**Affiliations:** 1 Department of Anatomical Sciences, School of Medicine, Iran University of Medical Sciences, Tehran, Iran; 2 Cellular and Molecular Research Center, Iran University of Medical Sciences, Tehran, Iran; 3 Reproductive Development, Murdoch Children's Research Institute, Melbourne, Victoria, Australia; 4 Department of Paediatrics, University of Melbourne, Melbourne, Victoria, Australia; 5 Department of Cellular Biotechnology, Cell Science Research Center, Royan Institute for Biotechnology, ACECR, Isfahan, Iran; 6 Shahid Akbarabadi Clinical Research Development Unit (SHACRDU), Iran University of Medical Sciences (IUMS), Tehran, Iran; 7 Department of Veterinary and Animal Sciences, Anatomy and Biochemistry Section, University of Copenhagen, Copenhagen, Denmark; 8 Department of Health and Medical Sciences, Faculty of Health, Arts and Design, Swinburne University, Hawthorn, Melbourne, Australia

**Keywords:** Granulosa Cell, Mitochondrial Biogenesis, Mitochondrial DNA, Polycystic Ovary Syndrome, Vitamin D3

## Abstract

**Background:**

Polycystic ovary syndrome (PCOS) is an endocrine disorder diagnosed by anovulation hyperandro- genism. Hyperandrogenism increases apoptosis, which will eventually disturb follicular growth in PCOS patients. Since mitochondria regulate apoptosis, they might be affected by high incidence of follicular atresia. This may cause infertility. Since vitamin D3 has been shown to improve the PCOS symptoms, the aim of study was to investigate the effects vitamin D3 on *mtDNA* copy number, mitochondrial biogenesis, and membrane integrity of granulosa cells in a PCOS-induced mouse model.

**Materials and Methods:**

In this experimental study, the PCOS mouse model was induced by dehydroepiandrosterone (DHEA). Granulosa cells after identification by follicle-stimulating hormone receptor (FSHR) were cultured in three groups: 1. granulosa cells treated with vitamin D3 (100 nM for 24 hours), 2. granulosa cells without any treatments, 3. Non-PCOS granulosa cells (control group). Mitochondrial biogenesis gene (TFAM) expression was compared between different groups using real-time PCR. *mtDNA* copy number was also investigated by qPCR. The mitochon- drial structure was evaluated by transmission electron microscopy (*TEM*). Hormonal levels were measured by an enzymelinked immunosorbent assay (ELISA) kit.

**Results:**

The numbers of pre-antral and antral follicles increased in PCOS group in comparison with the non-PCOS group. Mitochondrial biogenesis genes were downregulated in granulosa cells of PCOS mice when compared to the non-PCOS granulosa cells. However, treatment with vitamin D3 increased *mtDNA* expression levels of these genes compared to PCOS granulosa cells with no treatments. Most of the mitochondria in the PCOS group were spherical with almost no cristae. Our results showed that in the PCOS group treated with vitamin D3, the *mtDNA* copy number increased significantly in comparison to PCOS granulosa cells with no treatments.

**Conclusion:**

According to this study, we can conclude, vitamin D3 improves mitochondrial biogenesis and membrane integrity, mtDNA copy number in granulosa cells of PCOS mice which might improve follicular development and subsequently oocyte quality.

## Introduction

Polycystic ovary syndrome (PCOS) is a common endocrine disorder in women (1). Menstrual dysfunction, anovulation, hyperandrogenism, hirsutism, and polycystic ovaries are considered as the symptoms of PCOS (2). Hyperandrogenism stimulates follicular atresia in granulosa cells via apoptosis. As a result, apoptosis and oxidative stress can interfere with follicular growth in women Received: 23/August/2019, Accepted: 05/January/2020 *Corresponding Address: P.O.Box: 1449614525, Department of Anatomical Sciences, School of Medicine, Iran university of Medical Sciences, Tehran, Iran Email: Shirazi.r@iums.ac.ir Royan Institute International Journal of Fertility and Sterility Vol 14, No 2, July-September 2020, Pages: 143-149 suffering from PCOS (3). Therefore, disruption of follicular growth in patients suffering from PCOS is related to granulosa cells apoptosis and oxidative stress caused by high production of reactive oxygen species (ROS). Since mitochondria can regulate apoptosis and ROS production, these organelles may be affected by high rates of follicular atresia in the PCOS patients (4). Changes in the mitochondrial function might cause insulin resistance, oxidative stress, hyperandrogenism, and glucose intolerance, leading to the appearance of PCOS symptoms (5-7). The disturbance of mitochondrial function in granulosa cells may cause some disorders in oocyte function, maturation, and fertilization. This may also affect the fertility of PCOS patients by reducing the oocyte quality. Therefore, the proper functionality of mitochondria is of importance in this regard (5). Mitochondria play a key role in the determination of numerous factors involved in the reproduction, such as oocyte quality, follicular growth, development, and granulosa cell proliferation (6). Mitochondria also mediate various cellular processes, including apoptosis, ROS production, calcium signaling, adenosine triphosphate (ATP) synthesis, pyrimidine synthesis, and Fe-S protein synthesis (3, 8). Furthermore, mitochondria, as the main organelle for cellular ROS production, can also impair mitochondrial DNA (*mtDNA*), which may subsequently be the cause of different diseases. Being more susceptible to oxidative damage and attaining high rates of mutations are more common in the mitochondrial genome than nuclear DNA due to the adjacency to the electron transport chain (ETC), lack of sheltering histones and inefficient DNA repair capabilities (9). mtDNA of mammals is nearly 16 kb in size, encrypting 13 proteins of the oxidative phosphorylation (OXPHOS) complexes, 22 ribosomal RNAs (*rRNA*), and transfer RNAs (*tRNA*) that are required for mitochondrial mRNA translation. *mtDNA*, like nuclear DNA, can influence mitochondrial gene expression, biogenesis, and function through epigenetic modifications (10). It is of note to mention that mitochondrial biogenesis can also influence mtDNA and nuclearencoded protein synthesis, the congregation of the double genetic origin derived proteins, *mtDNA* replication as well as cell growth and proliferation (11). Mitochondrial biogenesis is hard to understand and needs several processes, such as synthesis of *mtDNA* and nuclear genes (12). The main gene in mitochondrial biogenesis that is critical for *mtDNA* transcription and maintenance is mitochondrial transcription factor A (*TFAM*). Mitochondrial biogenesis is also regulated by nuclear genes such as NRF2, which controls the other factors in mitochondria (11).

Since oocyte quality is a crucial factor for conception in PCOS patients and that depends on mitochondrial function and structure, prescription of an appropriate medication may improve fertility rate as a consequence of improved oocyte quality (13). Different treatments have been trialed, and vitamin D3 is one of which to have shown signs of improvement in PCOS patients (6). Vitamin D3 has been used before to alleviate signs of insulin resistance, hyperandrogenism, and oxidative stress in PCOS patients and other metabolic disorders (14). Vitamin D3 also has an important role in calcium homeostasis, cellular proliferation, and differentiation(15). Recently it has been demonstrated that the low level of vitamin D can result in excessive androgen secretion, insulin resistance, and follicular growth interruption in the patients suffering from PCOS. These occur through the decline of sex hormone-binding globulin (SHBG) levels, insulin receptors, and calcium dysregulation (8). The serum concentration of 25-hydroxyvitamin D in women with PCOS is less than 20 ng/ml, which can exacerbate PCOS symptoms (16). Therefore, in this study, we aimed to investigate the effects of vitamin D3 on the mitochondrial biogenesis, membrane integrity, and *mtDNA* copy numbers in the granulosa cells isolated from PCOS-induced mice.

## Materials and Methods

### PCOS animal model and assessment of morphology

This is an experimental study that the effect of vitamin D3 on mitochondrial biogenesis in a PCOS mouse model was investigated. Androgen excess and other symptoms of PCOS were induced by the injection of DHEA (Sigma, Austria), 6 mg/100 g body weight. DHEA was dissolved in 95% ethanol (0.01 mL) and mixed with sesame oil (0.09 mL). Subsequently, it was injected subcutaneously into female BALB/C mice (25 days old) for 20 consecutive days before reaching puberty (PCOS group, n=20). As a vehicle control, 0.1 mL of sesame oil (Sigma, Austria) and 0.01 mL of 95% ethanol (Sigma, Austria) were injected into another group of the same mouse strain for 20 consecutive days (n=20). A Control group of the same mouse strain without any treatment was also considered (n=20). The mice were kept at room temperature (25 ± 1°C, RT), with enough food and water, and under diurnal modulation by daily light. All the animal trials were performed in agreement with the Institutional Animal Care Committee of Iran University of Medical Sciences and Health Services for animal welfare. (ethics code: IR.IUMS.REC 1396.29969). The weight changes in mice were measured every day. Vaginal smears were also taken every day over the 20-day course of treatment. The mice were sacrificed by cervical dislocation. For histological assessments, the ovaries were subsequently fixed with 10% formalin (Merck, Germany). Next, 5-μm sections were made with a microtome, and the sections were immersed in xylene and ethanol with different grades for deparaffinization and rehydration, respectively (Merck, Germany). The ovaries were then stained with hematoxylin and eosin (DAKO, USA). For morphology assessment, the ovaries assessed by a Nikon microscope (Nikon, Japan), and photographs were taken.

### Sex hormones assessments

For the analysis of sex hormones, cardiac blood samples were collected using needles. Blood serum was subsequently separated using a centrifuge machine at (300 rpm, 4°C, 10 minutes) and follicle-stimulating hormone (FSH), luteinizing hormone (LH), 17β-estradiol (E2) and progesterone levels were measured by an ELISA kit (Abcam, Cambridge, UK) according to the manufacturer's guidelines.

### Isolation and culture of granulosa cells

The ovaries of 45-day BALB/C mice (DHEA-reated and the vehicle group) were removed after the mice were sacrificed via cervical dislocation. For aspiration of the follicles, 25-gauge needles were used, and the follicles were aspirated in a solution made of phosphate buffer saline (PBS) and 1.0% bovine serum albumin (BSA) (Invitrogen, USA). 70-μm cell strainers (BD Falcon, MA, USA) were used to isolate granulosa cells from the other cells and tissues. Subsequently, granulosa cells were separated from the oocytes with a 40-μm cell strainer (BD Falcon, MA, USA). Blood cell contamination was removed by RBC lysis buffer after centrifugation at 1000 rpm (4°C, 10 minutes). Then, the pellet was mixed with phenol red-free DMEM/F12 medium containing 10% fetal bovine serum (Sigma, Austria). The medium was centrifuged at 1000 rpm (4°C, 10 minutes). Next, the pellet was removed and transferred to cell culture dishes containing DMEM-F12, 10% FBS (Sigma, Austria), 100 mg/ mL streptomycin (Sigma, Austria), 100 IU/mL penicillin (Sigma, Austria), 2 mM glutamine (Sigma, Austria), 1 mM sodium pyruvate (Sigma, Austria). The culture dishes were then incubated at 37°C, with 5% CO2 and 95% humidity.

### Identification of isolated GCs

To identify granulosa cells, an antibody against FSHR, a specific marker of granulosa cells, was used. Affinitypurified rabbit anti-follicle stimulating hormone receptor (FSHR) polyclonal antibody was purchased from antibodies-online (ABIN1872743). First, the cells were spread on a slide using a cytospin centrifugation device, and the slides were then immersed in a cold normal buffered formalin (NBF) solution to be fixed. Subsequently, the cells were washed with PBS and blocked using PBSTriton/BSA. Afterward, the primary FSHR antibody was added to granulosa cells overnight. The following morning the cells were washed three times with PBS and were subsequently treated with the secondary FSHR antibody for 30 minutes. DNA was counterstained with 4′,6-diamidino-2-phenylindole (DAPI). Preparations were washed in PBS before mounting on glass slides. Slides were viewed on an epifluorescence microscope and captured with a digital camera.

### Experiment design

The treatment groups for granulosa cells were as follows:

1. PCOS granulosa cells treated with vitamin D3 (100 nM) for 24 hours (17, 18)
2. PCOS granulosa cells without any treatments
3. Non-PCOS granulosa cells (control group)

### Reverse transcription- polymerase chain reaction and quantitative reverse transcription- polymerase chain reaction

The Trizol reagent (Sigma, Austria) was used to extract the total RNA of granulosa cells in all groups. Then, chloroform was added to the mixture of granulosa cells and the Trizol reagent. Afterward, the mixture was centrifugated at 1000 rpm (4 C, 10 minutes). The upper phase containing the total RNA was collected. Next, the total RNA was washed with 75% ethanol, allowed to air dry, and then reconstituted in diethylpyrocarbonate (DEPC) water. Using a cDNA synthesis kit (Thermo Scientific, USA), the total RNA was reverse-transcribed according to the manufacturer’s guideline. In summary, a mixture of the random hexamer, first-strand buffer (all from Fermentas), DNase- (Fermentas Inc, MD, USA) treated RNA, RiboLockTM RNase inhibitor, dNTP Mix, Dithiothreitol (0.1M) and SuperScriptTM II Reverse Transcriptase was made for reverse transcription of each sample. The thermocycler (company) was set at 25°C for 10 minutes, 43°C for 40 minutes, and 75°C for 15 minutes. Quantitative PCR was performed using 1 μl of cDNA in a reaction consisting of ROXTM Reference Dye, SYBR Premix EX TaqTM (Takara, Japan), and 1 μl of the desired primer. The β-actin gene was utilized as a housekeeping gene. The reactions were amplified with StepOne™ Real- Time PCR System (Applied Biosystems, MA, USA) as following: denaturation at 95 °C for 10 seconds, 35 cycles of amplification (95°C for 5 seconds and 60°C for 30 seconds), separation stage at 95°C for 15 seconds, 60°C for 1 minutes, and 95°C for 15 seconds. Using oligo 7.60 software to design primers. The TFAM forward primer was CCG AGC TCC TCC TCC TTT GC and the TFAM reverse primer was CCT ACA ACG CAG CGA CCG AG.

### Mitochondrial DNA quantification

For the measurement of the *mtDNA* copy number, quantitative polymerase-chain-reaction (qRT-PCR) was used. Forward primer and reverse primer were used to analyze *mtDNA*. SYBR Green I Master Mix (10 μl) (Sigma, Austria), which contains 10 pmol of reverse primer and 10 pmol of forward primer was mixed with DNA (10 ng). The qPCR set-up consisted of 4 segments: 50°C for 2 minutes, 95°C for 10 seconds followed by 40 cycles of denaturation at 95°C for 5 seconds, annealing at 59°C for 35 seconds, and extension at 72°C for 1 minutes. For each qPCR reaction, the copy number of the *mtDNA* and the threshold cycle number (Ct) of the β-actin gene were measured. The runs were replicated at least two times, and the normalization was performed against the housekeeping gene, β-actin. For the quantification of the *mtDNA* copy number, the double delta Ct analysis was applied.

### Electron microscopy of mitochondria

Isolated granulosa cells were fixed using 2.5% glutaraldehyde in PBS and then treated with 1.0% osmium tetroxide in the same buffer for the post-fixation procedure. For performing the dehydration process, ethanol and propylene oxide were used. Then inserted in epoxy resin, and sectioned. Using ethanolic uranyl acetate to contrast the sections and lead citrate and observed under a transmission electron microscope (Zeiss LEO 906 (TEM), 100 kV, Germany).

### Statistical analysis

The data in this experiment are expressed as the means and standard error of the mean (19) for three independent biological replicates. Statistical significance between different groups was evaluated and analyzed by on-way analysis of variance (ANOVA), followed by Tukey’s post hoc test. The level of statistical significance was set at P<0.05. The SPSS software (version 21.0) was utilized for the data analysis.

## Results

### Hormonal levels and cycle alteration in mice

In the PCOS group induced by DHEA, the serum level of estradiol and LH were higher when compared to control and vehicle groups, respectively (Table 1). The lower amount of FSH serum was detected in the PCOS group versus the control group and vehicle group due to estradiol negative feedback. The ratio of LH/FSH was significantly increased in the PCOS group in comparison to the vehicle group (Table 1). The estrous cycle was irregular in the PCOS group and ultimately stopped, whereas, in the control and vehicle group, normal cycles (nearly 5-7 days) continued as normal. Using mice in the control and vehicle groups that were just in the estrous cycle to exclude the influence of the estrous cycles on other measurements.

**Table 1:** Hormonal levels


Hormone	Control	Vehicle	DHEA

Estradiol (pg/mL)	132 ± 9.10	142 ± 8.52	3786 ± 13.1^**^
FSH (IU/L)	6. 59 ± 0.82	6.84 ± 0.29	4.11 ± 0.64
LH (IU/L)	5.60 ± 0.11	6.13 ± 0.38	18.58 ± 0.82^*^
LH/FSH (IU/L)	0.84 ± 0.13	0.89 ± 1.31	4.52 ± 1.28^**^
Progesterone (pg/mL)	3.647 ± 0.69	3.268 ± 0.54	2.369 ± 0.19


Data are presented as mean ± SD. *; P<0.05, **; P<0.005, FSH; Follicle-stimulating hormone, LH; Luteinizing hormone, DHEA; dehydroepiandrosterone.

### Histological analysis for characterization of PCOS ovaries

**Fig 1:**
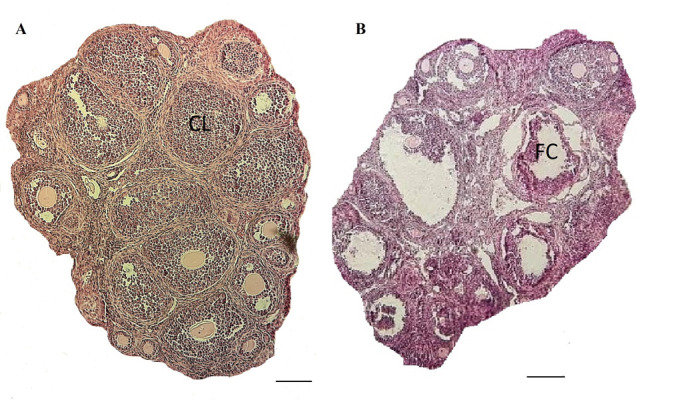
Histological assessment of ovaries. A. Follicles of normal ovaries represented follicles at different stages, and corpus luteum (CL) and B. Ovaries of the polycystic ovary syndrome (PCOS) model induced by dehydroepiandrosterone (DHEA) revealed antral and pre-antral follicles and some cysts (FC) were observed in H&E staining. No corpus luteum was observed in the PCOS ovary .Scale bar: 50 μm.

Upon H&E staining, the specimen was analyzed under the light microscope. Normal follicles were detected at various developmental stages in the vehicle group. Corpus luteum was also observed in the control group, which was an indicator of normal ovulation (Fig .1A). Due to the seizure of the estrus cycle, no corpus luteum was detected in the PCOS group (Fig .1B).

### Mitochondrial biogenesis gene expression

**Fig 2:**
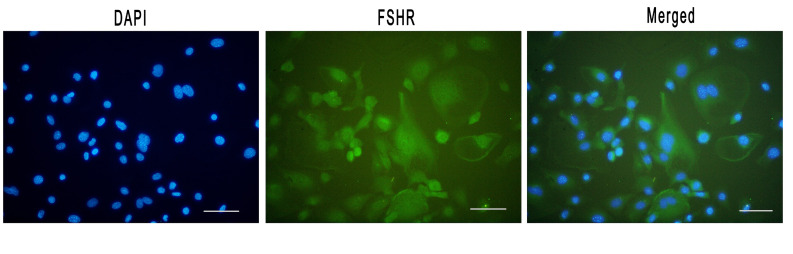
Follicle-stimulating hormone receptor (FSHR) (specific markers of granulosa cells) was investigated. The FSHR expression in isolated granulosa cells (green) was observed. Nuclei (blue) were stained by 4′,6-diamidino-2-phenylindole (DAPI). Scale bar: 100 μm.

**Fig 3:**
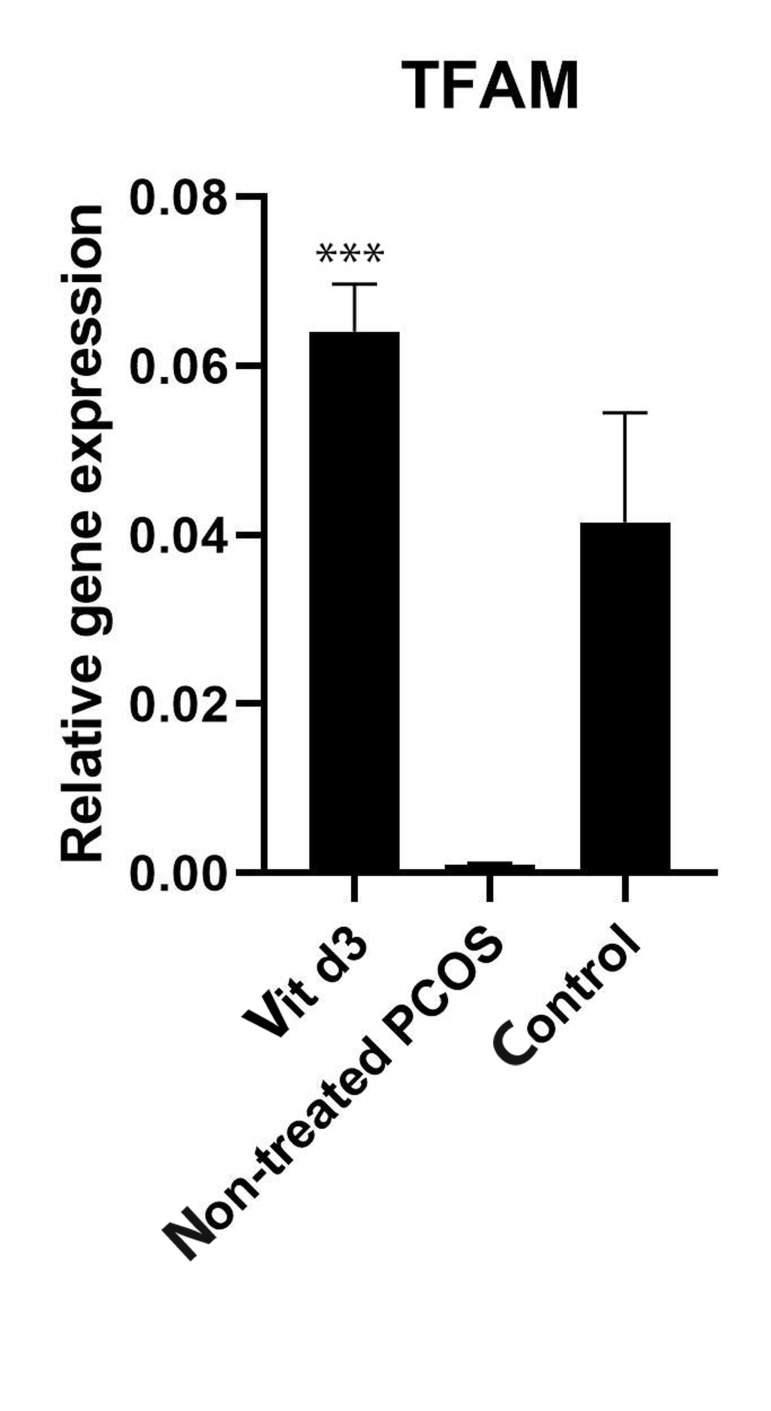
The expression of TFAM (mitochondrial biogenesis gene) in cultured granulosa cells of DHEA-induced PCOS BALB/C mice was compared between three groups. Granulosa cells were pre-incubated in the serumfree medium in the presence or absence of vitamin D3. The expression of the mitochondrial biogenesis gene was upregulated in the vitamin D3 group. It is also revealed that the gene expression was declined in PCOS granulosa cells in comparison with non-PCOS healthy granulosa cells (control group), ***; P<0.05, DHEA; Dehydroepiandrosterone, and PCOS; Polycystic ovary syndrome.

To assure that the cells being experimented on granulosa cells, a granulosa cell antibody was used against FSHR. Photograph analysis showed that the target cells were stained with this antibody, indicating that they were granulosa cells (Fig .2). Thus, for the treatment of granulosa cells, vitamin D3 (100 nM) was used for 24 hours. Subsequently, RNA was extracted, and Reverse transcription- polymerase chain reaction (RT-PCR) performed to measure the expression of mitochondrial biogenesis gene (TFAM) in different groups. Vitamin D3 increased the expression of TFAM in the PCOS group (Fig .3) by 5-fold compared to the PCOS group without any vitamin D3 treatment. This indicates that vitamin D3 might stimulate mitochondrial biogenesis in PCOS-induced granulosa cells.

### Mitochondrial DNA

**Fig 4:**
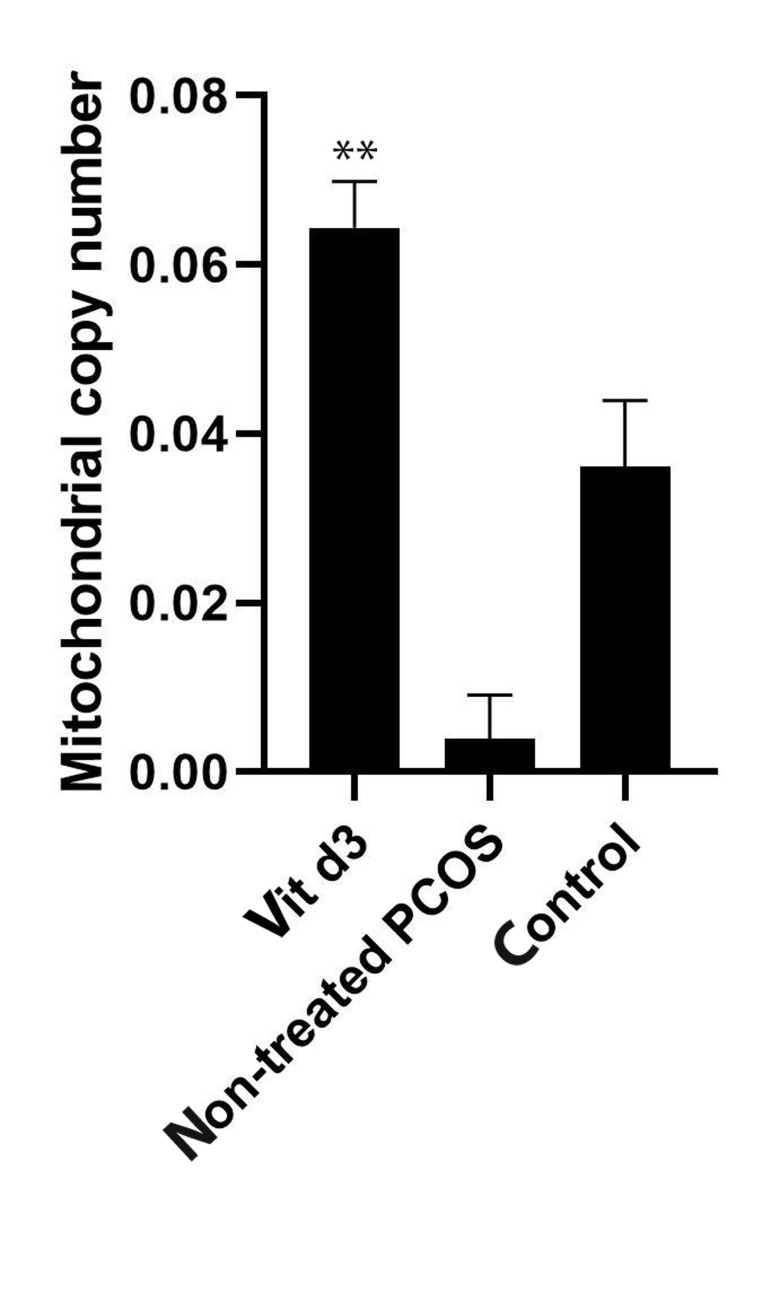
The mitochondrial DNA copy number (mtDNA) in cultured granulosa cells of DHEA-induced PCOS BALB/C mice was compared between three groups. Granulosa cells were pre-incubated in the serum-free medium in the presence or absence of vitamin D3. The mitochondrial DNA copy number was significantly increased in the vitamin D3 group in comparison with the non-treated PCOS group (**; P<0.05). It is also revealed that the mitochondrial DNA copy number was declined in the non-treated PCOS granulosa cells in comparison with the non-PCOS healthy granulosa cells (control group). DHEA; Dehydroepiandrosterone, PCOS; Polycystic ovary syndrome.

For the analysis of the *mtDNA*, qPCR was performed. Our results revealed that in the PCOS group treated with vitamin D3, the *mtDNA* copy number increased significantly in comparison to the non-treated PCOS group (Fig .4). Data analysis by the quartile distribution of *mtDNA* copy number in the non-treated PCOS group showed an association between *mtDNA* copy number and PCOS risk.

### Transmission electron microscopy of mitochondria structure

**Fig 5:**
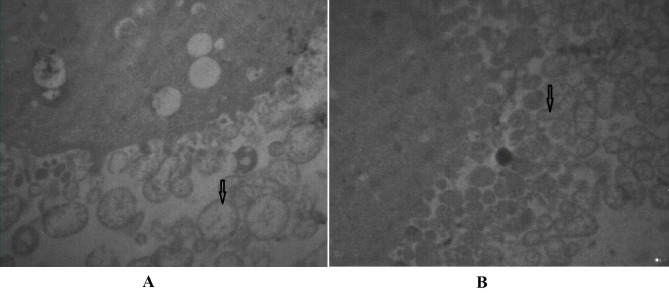
Mitochondria membrane structure (TEM). **A.** PCOS group without any treatments were spherical with almost no cristae. **B.** PCOS group treated with vitamin D3 and also in the non-PCOS group (control group) include undamaged mitochondria.

For the evaluation of the alterations of the mitochondria structure, transmission electron microscopy was employed. Most of the mitochondria in the PCOS group without any treatment were spherical, with almost no cristae; however, in the PCOS group treated with vitamin D3 as well as in the non- PCOS group (control group) intact inner and outer membrane and a clear intermembrane space was observed (Fig .5).

## Discussion

The present study demonstrated that vitamin D3 affected *mtDNA* copy number, mitochondrial structure, and mitochondrial biogenesis in granulosa cells of a PCOSinduced mouse model in comparison with healthy normal ovaries. PCOS is regularly described by oligomenorrhea, chronic anovulation, hyperandrogenism, and hyperinsulinemia (20). Androgenic hormones, such as DHEA, testosterone, and androstenedione, cause some problems in the patients suffering from PCOS (2). According to previous studies, in the PCOS ovaries, the atretic follicles increased that caused by hyperandrogenism, which is critical in the pathogenesis of PCOS. High levels of androgen in women that suffer from PCOS might intensify follicular atresia and follicular development disruption that might cause subfertility (4). According to our previous study (17), to mimic the hyperandrogenism condition, for induction of the PCOS model and also confirmation of the abnormal hormonal level and ovarian morphological features in PCOS mice, DHEA was injected into the 25- day old female mice intraperitoneally. Androgen excess, insulin resistance, and disturbed follicular development are some symptoms of this disorder that might interfere with female fertility (21-23). The levels of LH, estradiol, along with the ratio of LH to FSH were increased in the PCOS-induced mice compared to the vehicle group. The level of FSH was comparatively decreased in the PCOS group caused by the estradiol level feedback.

Disrupted ovulation and oocyte quality induced by hyperandrogenism can be improved by different treatments such as metformin and spironolactone (7). Besides hormonal treatments, various supplements such as vitamin D3 have been shown to improve the PCOS symptoms (24). Moreover, several lines of evidence demonstrate the positive role of vitamin D3 in some disorders, such as premature ovarian failure (POF), endometriosis, PCOS, and male infertility (17, 25). It has been demonstrated that vitamin D3 might stimulate follicular development in patients with PCOS; however, it could not alleviate disrupted lipid and glucose metabolism (26-28). A large body of studies has shown that vitamin D3 has constructive effects on alleviating the symptoms of ovulation disorders and insulin resistance in women suffering from PCOS disorder (17, 29). Hormonal fluctuations in PCOS women can be improved by vitamin D3; however, the duration of treatment can influence the degree of symptom alleviation (14, 26). Underlying mechanisms as to how vitamin D3 exerts its effects are yet to be elucidated.

For the assessment of the effect of vitamin D3 on mitochondrial biogenesis, isolated granulosa cells from PCOS ovaries were treated with vitamin D3. Mitochondrial membrane integrity and alteration in mtDNA copy numbers were also evaluated. It has been demonstrated that mitochondria, as the powerhouse of the cell, are of importance for optimum oocyte quality and fertilization. Poor oocyte quality and subsequent embryonic development could be attributed to mitochondrial dysfunction (10, 15). We hypothesized that vitamin D3 may improve *mtDNA* copy number, mitochondrial membrane integrity, and biogenesis.

In the present study, we demonstrated that mitochondrial biogenesis could be upregulated after 24 hours of treatment with vitamin D3. The findings showed that vitamin D3, as a supplementation, improves the main mitochondrial biogenesis marker (*TFAM*) in the granulosa cells of PCOS ovaries. According to some evidence, *TFAM* plays an important role in mitochondrial biogenesis (12, 30-33). It is revealed that total antioxidant capacity (TAC) raise by vitamin D3 and also vitamin D3 may alleviate the hormonal disturbances in women with PCOS (34, 35). Since our results showed that vitamin D3 has an improvement effect on ovulation problems and follicular disruption, we could understand that vitamin D3 might have an important role in declining the atretic follicles and alleviating the development of follicles via upregulating the mitochondrial biogenesis main gene and mitochondrial membrane integrity.

In this study, for the first time, we have shown the vitamin D3 effect on *mtDNA* copy number and mitochondrial membrane integrity in a mouse model of PCOS granulosa cells. Our results revealed that most of the mitochondria in the PCOS group were spherical with almost no cristae. In line with our study, Longfei et al. revealed a distorted mitochondrial structure and diminished membrane integrity in the PCOS group (10). Oocytes of the PCOS mouse model, induced with DHEA, have demonstrated disrupted mitochondrial biogenesis, decreased *mtDNA* copy number, and distorted mitochondrial ultrastructure that is in agreement with our findings in this study (10, 36). Ding et al. also observed that mitochondrial dysfunction, due to *mtDNA* mutation, has a role in the manifestation of PCOS symptoms that is in line with our results (37). Reduced *mtDNA* copy number is associated with poor oocyte quality and subsequent compromised embryo development and implantation (10) (15). Our findings showed decreased *mtDNA* copy number in the PCOS group (nontreated), which is increased upon treatment with vitamin D3

In line with this finding, researchers showed reduced mtDNA copy number in the PCOS patients (37, 38). Also, in agreement with our findings, a case-control study showed reduced *mtDNA* copy number in Korean women suffering from PCOS (9, 10, 39). Bhanoori et al. (9) also demonstrated that mtDNA copy number severely decreased in PCOS patients.

## Conclusion

According to our results, mtDNA copy number, the biogenesis might be affected by vitamin D3 in PCOS granulosa cells. We nominate that mitochondrial biogenesis genes expression might be increased by vitamin D3. Therefore, vitamin D3 can have a significant role in the alleviation of mitochondria and follicular damages in PCOS ovaries. However, extensive studies are needed to determine the optimal dose and duration of treatment with vitamin D3 in PCOS women.
